# Versatile nanoarchitectonics of Pt with morphology control of oxygen reduction reaction catalysts

**DOI:** 10.1080/14686996.2022.2088040

**Published:** 2022-06-22

**Authors:** Guoping Chen, Santosh K. Singh, Kotaro Takeyasu, Jonathan P. Hill, Junji Nakamura, Katsuhiko Ariga

**Affiliations:** a Graduate School of Frontier Sciences, The University of Tokyo, Kashiwa, Chiba, Japan; b International Center for Materials Nanoarchitectonics, National Institute for Materials Science, Tsukuba, Japan; c Faculty of Pure and Applied Sciences, Tsukuba Research Centre for Energy and Materials Science, University of Tsukuba, Tsukuba, Ibaraki, Japan

**Keywords:** Nanoarchitectonics, nanoparticles, oxygen reduction reaction, platinum

## Abstract

Electro-catalytic activity of Pt in the oxygen reduction reaction (ORR) depends strongly on its morphology. For an understanding of how morphology affects the catalytic properties of Pt, the investigation of Pt materials having well-defined morphologies is required. However, the challenges remain in rational and facile synthesis of Pt particles with tuneable well-defined morphology. A promising approach for the controlled synthesis of Pt particles is ‘self-assembly of building blocks’. Here, we report a unique synthesis method to control Pt morphology by using a self-assembly route, where nanoflower, nanowire, nanosheet and nanotube morphologies of Pt particles have been produced in a controlled manner. In the growth mechanism, Pt nanoparticles (5–11 nm) are rapidly prepared by using NaBH_4_ as a reductant, followed by their agglomeration promoted by adding 1,2-ethylenediamine. The morphology of the resulting Pt particles can be easily controlled by tuning hydrophobic/hydrophilic interactions by the addition of isopropanol and H_2_O. Of the Pt particles prepared using this method, Pt nanotubes show the highest ORR catalytic activity in an acid electrolyte with an onset potential of 1.02 V vs. RHE.

## Graphical abstract

**Figure:**
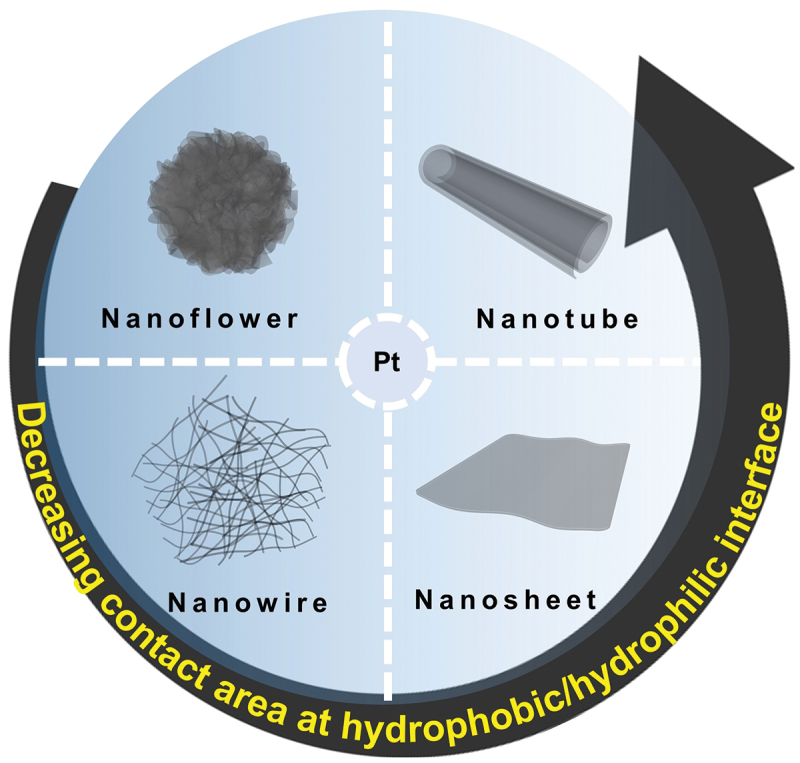

## 1. Introduction

Platinum (Pt) is the most effective catalyst for many catalytic processes including the oxygen reduction reaction (ORR), which occurs in proton exchange membrane fuel cells [1,2]. However, the high cost and relatively poor stability of Pt catalysts remain as obstacles to their widespread commercial use, while any improvements in their catalytic activity might reduce the quantities of the metal required for the operation of fuel cells [3]. It has been recently reported that ORR activity of Pt can be increased through the morphological control of its particles, that is, as nanowires [4], nanosheets [5], nanotubes [6], suggesting that the surface structure of Pt particles is significant in determining catalytic activity. While specific reasons for improvements in activity are not clear, there exist several factors including hydrophobic effects that prevent hydration of the reaction intermediates [7], or that increase the local concentration of O_2_ [8,9], and structural effects such as orientation dependence of Pt-surface planes [10]. To determine the importance of these effects, further studies of Pt particles having better defined morphologies, such as hierarchical Pt nanostructures integrating different components leading to unique properties and functionalities, is required [11]. For example, hollow Pt nanostructures containing multiple cavity and pore structures possess larger surface areas and increased numbers of catalytically active sites for ORR [12]. However, the challenges remain in rational and facile synthesis of Pt particles with tuneable well-defined morphology.

The conventional method for synthesis of Pt particles involves a chemical reduction method, where Pt precursors, such as PtCl_2_, H_2_PtCl_4_ or H_2_PtCl_6_ are treated in solution using reductants such as sodium borohydride (NaBH_4_), lithium aluminum hydride (LiAlH_4_) or hydrazine (N_2_H_4_) [2,13]. Additives such as surfactants, polymers, halide ions, or amines are sometimes used to control surface properties and affect the morphologies of the resulting Pt particles [14,15]. These additives act as capping agents, which interact non-uniformly with the different crystal faces of Pt dictating shape evolution [16], regulate the kinetics of Pt particles formation and growth [17], and prevent the agglomeration of Pt particles [18]. These functions derive from adsorption of the capping agent at specific sites on the Pt surfaces where steric and/or electrostatic effects lead to sensitive modification of the local environment [19]. Widely used additives include poly(vinylpyrrolidone) (PVP), oleyl amine and sodium citrate. For example, Huang et al. synthesized octapod polyhedral Pt particles using methylamine and PVP, where PVP disperses Pt particles [20].

Despite the importance (and consequent in depth investigations) of the effects of additives on the shapes of Pt particles, the mechanisms of control are poorly understood, and there remain significant challenges to the rational and facile synthesis of Pt catalysts with tunable morphologies. A promising approach, which has emerged as an effective method for the preparation of well-defined superstructures of Pt particles, is ‘self-assembly of building blocks’ [21]. Self-assembly has led to several surprising insights into materials’ synthesis and is indeed a rewarding area of investigation [22,23]. The purpose of the work presented here has been to control morphology of Pt particles by taking advantage of the self-assembly approach. It has been widely reported that surfactants self-assemble establishing a soft template with subsequent deposition of Pt atoms at specific regions leading to replication of the form of the template [24,25]. For example, Song et al. employed a seeding/autocatalytic approach in micellar solution containing liposomes to synthesize a solid foam like Pt structure comprised of dendritic sheets, indicating a templating effect of the surfactant assembly [25]. However, these soft template self-assembly methods cannot be used to control the morphologies of Pt particles. Some reports have suggested that nanoparticles (NPs) can act as building blocks in a versatile nanoarchitectonics approach [26]. For example, Liao et al. have observed that Pt_3_Fe nanorods form from kinked chains of connected NPs that undergo reorientation and a straightened process [27]. Schliehe et al. have shown that chlorine-containing cosolvents can direct the colloidal synthesis of PbS NPs into 2D nanosheets [28]. In self-assembly processes, ligand molecules play a crucial role by selectively capping the surfaces of NPs, and can hinder, modify, or trigger an oriented attachment [29]. Despite this, little research, effort has been devoted to investigating the self-assembly behavior of Pt NPs as building blocks.

Here, we report a novel self-assembly method for the synthesis of Pt nanostructures of diverse morphology including nanoflowers (PtNFs), nanowires (PtNWs), nanosheets (PtNSs), and nanotubes (PtNTs) by taking advantage of several interactions, such as hydrogen bonding, hydrophilic–hydrophobic interface interactions occurring in the liquid phase. In this process, it is clear that Pt NPs initially formed in a rapid reduction reaction using NaBH_4_, are then agglomerated by introducing 1,2-ethylenediamine (EDA) to construct various morphologies of Pt by controlling hydrophobic/hydrophilic interactions through the addition of isopropanol (IPA) and water (H_2_O). Some of the resulting Pt nanostructures exhibited higher specific activity as ORR catalysts than commercial Pt black in acidic electrolyte.

## 2. Materials and methods

### 2.1. Materials

Chemicals used were of analytical grade and were used as received without further purification. Platinum (II) chloride (PtCl_2_), 1,2-ethylenediamine (EDA, 99%), sodium borohydride (NaBH_4_, 99.99%) and isopropanol (IPA, 99.7%) were purchased from Wako Pure Chemical Industries, Japan.

### 2.2. Preparation of Pt particles

#### 2.2.1. Synthesis of Pt nanoflower

In a typical procedure, PtCl_2_ (30 mg) was dispersed in H_2_O (2.5 mL) then mixed with EDA (7.5 mL) followed by sonication for 10 min to form the Pt/EDA precursor solution. NaBH_4_ (20 mg) was dissolved in H_2_O (0.3 mL) then mixed with Pt/EDA (2 mL) precursor solution. After heating the reaction mixture at 100°C for 40 min in a vessel open to the air, the powder product was collected by centrifugation. Pt nanoflower was obtained as the product after twice washing with deionized H_2_O.

#### 2.2.2. Synthesis of Pt nanowire

Isopropanol (3.4 mL) was added to the above Pt/EDA precursor solution (2 mL) to form Pt/EDA/IPA precursor solution. NaBH_4_ (20 mg) was dissolved in H_2_O (0.3 mL) then mixed with the Pt/EDA/IPA precursor solution. After heating the reaction mixture at 100°C for 40 min in a vessel open to the air, the powder product was collected by centrifugation. Pt nanowire was obtained after twice washing with deionized H_2_O.

#### 2.2.3. Synthesis of Pt nanosheet

Isopropanol (3.4 mL) was added to the above Pt/EDA precursor solution (2 mL) to form Pt/EDA/IPA precursor solution. NaBH_4_ (20 mg) was dissolved in H_2_O (0.5 mL) then mixed with the Pt/EDA/IPA precursor solution. After heating the reaction mixture at 100°C for 40 min in a vessel open to the air, the powder product was collected by centrifugation. Pt nanosheet was obtained after twice washing with deionized H_2_O.

#### 2.2.4. Synthesis of Pt nanotubes

Isopropanol (3.4 mL) was added to the above Pt/EDA precursor solution (2 mL) to form Pt/EDA/IPA precursor solution. NaBH_4_ (20 mg) was dissolved in H_2_O (0.7 mL) then mixed with the Pt/EDA/IPA precursor solution. After heating the reaction mixture at 100°C for 40 min in a vessel open to the air, the powder product was collected by filtration. Pt nanotubes were obtained after twice washing with deionized H_2_O.

### 2.3. Characterization

The morphology of the products was characterized by scanning electron microscopy (SEM, S-4800, Hitachi Co. Ltd Tokyo Japan) at an accelerating voltage of 30 kV, and transmission electron microscopy (TEM, JEOL 2100F, Tokyo Japan) at an accelerating voltage of 200 kV. X-ray diffraction (XRD, RIGAKU MiniFlex 600, Japan) patterns were collected using Cu Kα radiation (λ = 1.5418 Å). X-ray photoelectron spectroscopy (XPS, JPS 9010TR, JEOL, Japan) using Mg Kα radiation was used to analyse the chemical state of powdered samples. Metrohm Autolab potentiostat-galvanostat (PGSTAT302N, Metrohm, Japan) was employed for the electrochemical measurements.

### 2.4. Electrochemical measurements of ORR

For the electrochemical ORR characterization of the catalysts, a catalyst ink was prepared by dispersing each catalyst (5 mg) in IPA (580 µL) and water (380 µL). To the resulting dispersed solution. Nafion® solution (5% w/v, 40 µL) was added, followed by sonication for 15 min.  The resulting well-dispersed catalyst ink solution was stored prior to electrode preparation.

Prior to coating using the catalyst ink, the glassy carbon disc electrode was polished by employing the electrode polishing kit procured from ALS Co. Ltd. Catalyst ink (30 µL) was then dropped onto the polished RDE electrode followed by drying at room temperature. Electrochemical measurements were carried out by employing catalyst coated electrode as working electrode (WE), platinum wire as counter electrode (CE), and reversible hydrogen electrode (RHE) as reference electrode (RE) under N_2_/O_2_ saturated 0.1 M H_2_SO_4_ as electrolyte. The cyclic voltammogram (CV) measurement for the electrocatalytic surface area was carried out at a scan rate of 10 mV s^−1^ under N_2_-saturated 0.1 M H_2_SO_4_ without rotation of the WE. Linear sweep voltammogram (LSV) was recorded at the scan rate (10 mV s^−1^) under N_2_/O_2_ saturated electrolyte solution with rotation of WE at 1600 revolutions per minute (RPM). The LSV for the ORR is presented after the subtraction of the LSV current obtained under N_2_ from the O_2_ LSV current (*I*_O2_-*I*_N2_). ORR current density was obtained by normalizing the achieved current with the electrode area.

## 3. Results and discussions

### 3.1. Characterization of the Pt particles

**Figure 1.**
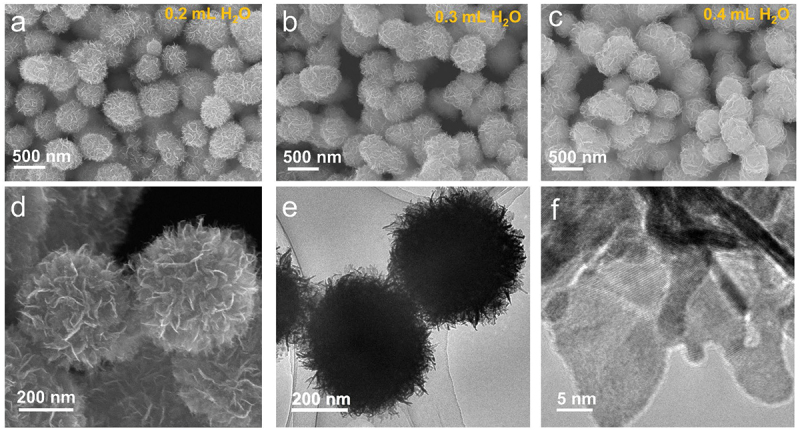
SEM images of synthesized Pt nanoflowers at condition of (a) 0.2 mL H_2_O, (b) 0.3 mL H_2_O and (c) 0.4 mL H_2_O. (d) Electron microscopy of synthesized Pt nanoflowers at condition of 0.3 mL H_2_O: (d) Magnified SEM image and (e, f) TEM images.

The formation of PtNFs was observed by a simple process involving the Pt precursor (PtCl_2_), reductant (NaBH_4_), and connecting reagent (EDA). In a typical PtNFs synthesis, Pt/EDA precursor solution (3 mg mL^−1^) was prepared by dispersing PtCl_2_ in deionized H_2_O then mixed with EDA (H_2_O/EDA = 25/75 v/v). Aqueous NaBH_4_ solution was prepared by dissolving NaBH_4_ (20 mg each) in 0.2, 0.3, and 0.4 mL of H_2_O. Pt/EDA precursor solution (2 mL) was then added to each of these aqueous NaBH_4_ solutions. After heating the reaction mixture at 100°C for 40 min (open to air), the powder product was collected and washed twice with deionized water. Figure 1 shows scanning electron microscopy (SEM) and transmission electron microscopy (TEM) images of PtNFs. The SEM images in Figure 1(a–c) reveal the nanospherical morphology of PtNFs synthesized in 0.2, 0.3 and 0.4 mL H_2_O, respectively. The morphologies of the resulting PtNFs are similar regardless of the volume of H_2_O used, having thin ‘petals’ and diameters around 500 nm. Figure 1(d–f) show magnified SEM and TEM images of PtNFs synthesized in 0.3 mL H_2_O and clearly indicate that PtNFs are assemblies of ultrathin nanosheets. The crystalline particle size of PtNFs lies in the range 3-15 nm.

**Figure 2.**
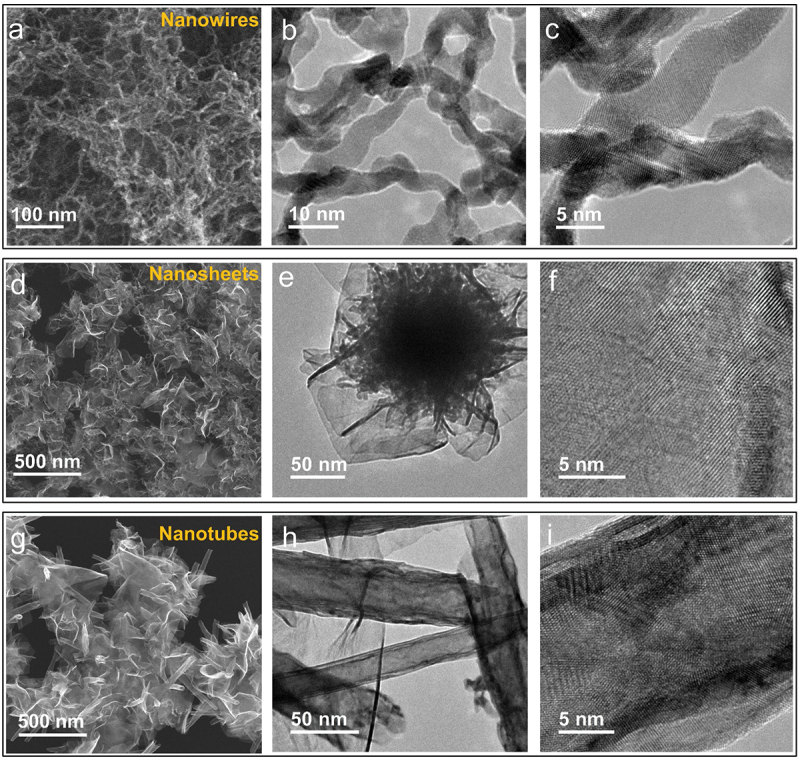
Electron microscopy of Pt nanostructures. Pt nanowires: (a) SEM image and (b, c) TEM images; Pt nanosheets: (d) SEM image, (e, f) TEM images; Pt nanotubes: (h) SEM image and (h, i) TEM images.

Figure 2 shows SEM and TEM images of PtNWs, PtNSs, and PtNTs. When IPA was added to the Pt/EDA precursor at a ratio of EDA: IPA = 1:2 to prepare a Pt/EDA/IPA precursor solution, the formation of PtNWs, PtNSs and PtNTs was observed depending on the volume of H_2_O added. Aqueous NaBH_4_ solution was prepared by dissolving NaBH_4_ (20 mg each) in 0.3, 0.5, and 0.7 mL of H_2_O. Aliquots (5.4 mL) of the Pt/EDA/IPA precursor solution were then added to each of these aqueous NaBH_4_ solutions. The reaction mixtures were then placed on a hotplate at 100°C for 40 min (open to air) to obtain PtNWs, PtNSs and PtNTs, respectively. The SEM image shown in Figure 2(a) reveals that the PtNWs obtained are composed of an ultrafine interconnected nanomesh structure. The TEM images (Figure 2(b,c)) indicate that PtNWs have diameters around 5 nm. The SEM image in Figure 2(d) shows the two-dimensional morphology of PtNSs with TEM images (Figure 2(e)) further confirming the sheet-like structure. Their high transparency and wrinkled form further indicate an ultrathin structure for PtNSs. Magnified TEM images of a single nanosheet (Figure 2(f)) reveal that the nanosheets are highly crystalline. The SEM image shown in Figure 2(g) displays a novel nanotubular form of PtNTs, most likely formed by rolling-up of Pt nanosheets. Several non-curled or partially curled nanosheets can also be observed strongly supporting the rolling-up mechanism. The TEM images shown in Figure 2(h,i) confirm the tubular structure of Pt by the strong contrast between the dark edges and the more transparent centers. Diameters of the nanotubes lie in the range from 20 to 50 nm. To our knowledge, this is the first report of the formation of PtNT by rolling up of PtNS.

**Figure 3.**
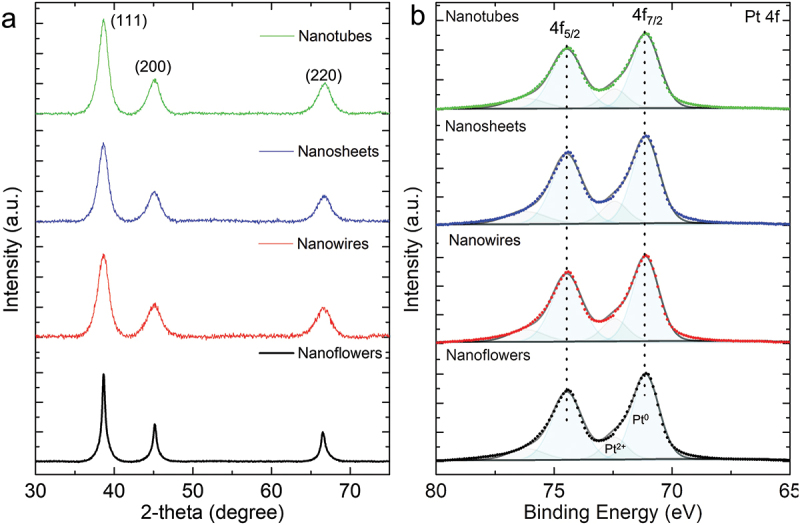
(a) XRD patterns and (b) XPS spectra of Pt 4f for Pt nanoflowers, Pt nanowires, Pt nanosheets and Pt nanotubes.

Figure 3(a) shows XRD patterns of PtNFs, PtNWs, PtNSs, and PtNTs, where peaks at 8.65, 45.15 and 66.52° correspond to the (111), (200) and (220) planes of Pt, respectively [2]. Average grain sizes of Pt NPs contained in the materials and estimated from the (111) peak, were found at 11.3 nm (PtNFs), 5.7 nm (PtNWs), 5.9 nm (PtNSs) and 5.1 nm (PtNTs), respectively. XRD results strongly suggest that Pt NPs with diameters of 5–11 nm are self-assembled at the early stages of PtNFs, PtNWs, PtNSs, and PtNTs formation. The XRD results are also consistent with TEM results in terms of the crystallite size of 5–15 nm. The difference in grain sizes of PtNFs and the other Pt particles (PtNWs, PtNSs and PtNTs) is attributed to the addition of IPA, which may lower the rate of reduction by NaBH_4_. We have calculated the values of the texture coefficients for (111), (200) and (220) planes of Pt particles (PtNFs, PtNWs, PtNSs, and PtNTs) normalized to those of Pt black as listed in Table. S1 [30]. The texture coefficients for (111) planes are distributed between 1.12 and 1.19 for all the Pt particles whereas the texture coefficients for (220) planes are below 0.87. This indicates that the (111) plane is preferred in Pt particles compared to Pt black and the ratios of (110) facets are smaller.

XPS analysis was used to characterize the chemical states of the Pt structures, and the results are shown in Figure 3(b). Pt 4f spectra of all the samples contain two asymmetric peaks (Pt 4f_7/2_ and Pt 4f_5/2_, respectively), indicating different states of Pt. Main peaks located at 71.1 and 74.4 eV are attributed to metallic Pt. shoulder peaks observed at higher binding energy can be assigned to Pt^2+^, which might be due to exposure of the samples to the atmosphere [31–33].

### 3.2. Study of the catalytic properties of the Pt particles

**Figure 4.**
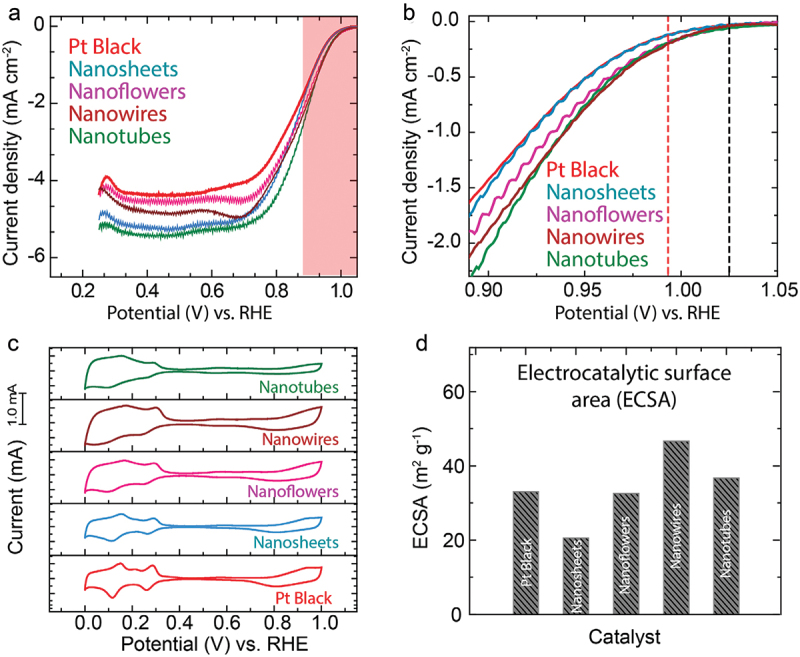
Electrocatalytic activity of the nanostructured Pt catalysts. (a) LSV recorded at a scan rate of 10 mV s ^−1^ and 1600 RPM under O_2_-saturated 0.1 M H_2_SO_4_; (b) enlarged LSV showing the *E*_onset_. (c) Comparison of CV of the catalysts at 10 mV s ^−1^ scan rate. (d) Relative ECSA of the catalysts.

The electro-catalytic activities of the prepared Pt particles were evaluated in a conventional 3-electrode set-up comprising a rotating disc electrode coated with the Pt material as catalyst (working electrode, WE), platinum wire (counter-electrode, CE), and reversible hydrogen electrode (RHE) in N_2_/O_2_ saturated 0.1 M H_2_SO_4_ electrolyte. The ORR activities of the catalysts were evaluated at least three independent experiments, and the data are shown in Figure S1. Figure 4(a,b) show the linear sweep voltammograms (LSV) for ORR using the Pt particle catalysts. LSV for the ORR is presented after the subtraction of the LSV current obtained under N_2_ from that obtained under O_2_ (*I*_O2_-*I*_N2_). The differences in the limiting current densities are probably due to the roughness and inhomogeneity of the electrode surface [34]. An important indicator for the commercialization potential of Pt catalysts is their mass activities (MA). As shown in Table 1, all of the Pt particle catalysts exhibit improved mass activities relative to Pt black. The enhancements in mass activity of Pt NTs, Pt NFs and Pt NSs can be attributed to improvements in specific activity and excellent particle dispersion. The small dimensions of Pt NWs might also be responsible for improvements in mass activity. While mass activity is an important parameter, it is also necessary to consider the specific activity to assess the effects of surface structure on Pt on ORR activity.

**Table 1.** Comparison of loading, electrocatalytic surface areas (ECSA), current@ 0.9 V, mass activity (MA) and specific activity (SA) of Pt catalysts.

Catalyst	Loading (mg)	ECSA (m_pt_^2^ g^−1^)	Current@ 0.9V (mA)	MA@ 0.9V (A g^−1^)	SA@ 0.9V (A m_pt_^−2^ 10^−2^)
Pt-black	0.15	33.1	0.41 ± 0.04	2.7 ± 0.3	8.3 ± 0.8
PtNSs	0.15	20.6	0.42 ± 0.04	2.8 ± 0.3	13.6 ± 1.3
PtNFs	0.15	32.6	0.49 ± 0.03	3.3 ± 0.2	10.0 ± 0.6
PtNWs	0.15	46.7	0.51 ± 0.04	3.4 ± 0.3	7.3 ± 0.6
PtNTs	0.15	36.7	0.57 ± 0.04	3.8 ± 0.3	10.4 ± 0.8

In order to examine the differences in ORR activity of the Pt catalysts, we conducted cyclic voltammetry (CV) under N_2_ saturated aq. H_2_SO_4_ electrolyte at a scan rate of 10 mV s^−1^ as shown in Figure 4(c). It is known that the form of the CV response is dependent on Pt surface structure so that CV gives us specific information regarding atomic surface structures of the Pt particles [35–37]. The electrocatalytic surface areas (ECSA) and specific activities (SA) calculated at 0.90 V of the Pt particle catalysts were listed in Table 1. Interestingly, PtNFs, PtNTs, and PtNWs catalysts have larger ECSA than Pt NSs (Figure 4(d)). This is because the sheet structure is composed of stacked Pt layers. Therefore, compared to the PtNFs, PtNTs, and PtNWs with smaller scale stacked structures, the access of ions to the internal layers is restricted for Pt NSs, leading to a decrease in ECSA. The highest ECSA of Pt NWs is expected based on its low dimensions (5 nm). For Pt(111), H-adsorption (Pt + H^+^ + e^-^ ➔ Pt-H_ads_) and desorption (Pt-H_ads_ ➔ Pt + H^+^ + e^-^) peaks (H_upd_ peaks) are distributed in the range 0.0 to 0.5 V vs. RHE and are more intense at lower potentials compared to Pt(110) and Pt(100) crystal planes [35,36]. The data show that the CV responses of PtNFs, PtNTs, and PtNWs catalysts resemble more closely that of Pt(111) than that of Pt(110). The texture coefficient values in Table S1 also show that the fractions of Pt(111) in Pt particles are higher than that of Pt black. It should be pointed out that the order of ORR activity in aqueous H_2_SO_4_ is Pt(110) > Pt(100) > Pt(111) [38] or Pt(100) > Pt(110) > Pt(111) [39]. However, the specific activities are higher for PtNSs, PtNTs, and PtNFs, as shown in Table 1. This suggests that differences in ORR activity are not due simply to the preferred planes of Pt but have another origin.

There are several possible reasons for the observed differences in specific activity. Local hydrophobicity caused by a hierarchically rough surface can affect ORR activity, which may be one of the reasons [40]. This feature has also been invoked to explain disruption of coverage of the water layer at a rough Pt surface [41]. In this work, a hierarchical structure of nanosheets was observed for both Pt NFs and Pt NSs, while a curved nanotubes structure was observed for Pt NTs. Thus, it is reasonable to expect improved activity based on these structural features. Additionally, we speculate that smaller hierarchically structured particles possess a larger total area of locally hydrophobic regions. Pt NFs have dimensions around 500 nm, while the particle size of Pt NSs is less at 200–300 nm caused by the addition of IPA during synthesis. As the H_2_O content increases, the size of the Pt NTs increases compared to the Pt NSs to reduce the specific surface energy. This leads to higher specific activity for Pt NSs over Pt NFs and Pt NTs, and also accounts for the comparable specific activities of Pt NFs and Pt NTs.

Morphological control of Pt particles has been achieved here by applying a novel nanoarchitectonic approach. It is promising to note that the controlled morphology (nanoarchitectonic structure) is directly associated with the macroscopic properties, which is in turn reflected in the improved ORR activities of the Pt catalysts [42].

### 3.3. Possible mechanism of Pt particle formation

**Figure 5.**
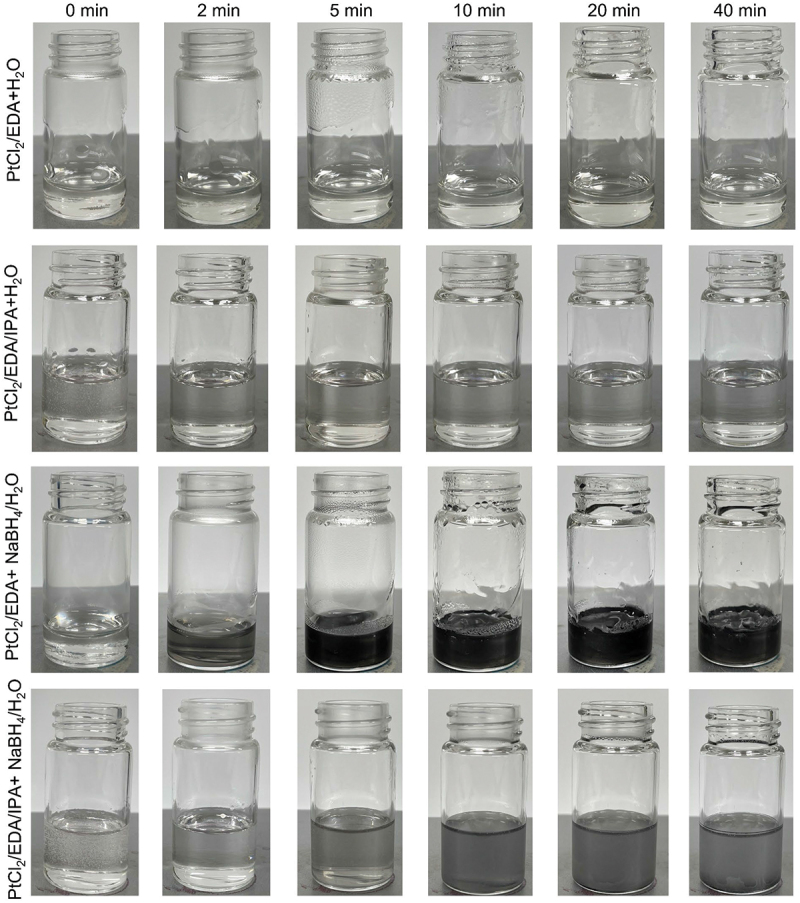
Digital images of different reactions in bottles at 100℃ with reaction time of 0, 2, 5, 10, 20 and 40 min, respectively, in which the volume of PtCl_2_/eda precursor is 2 mL, the volume of H_2_O is 0.3 mL, the volume of IPA is 3.4 mL, and the amount of NaBh_4_ is 20 mg.

To understand the role of NaBH_4_ in this work, we have carried out experiments to track the formation of Pt particles at 100°C at reaction times of 0, 2, 5, 10, 20 and 40 min, as shown in Figure 5. In the absence of NaBH_4_, the solutions (PtCl_2_/EDA + H_2_O and PtCl_2_/EDA/IPA + H_2_O) remain clear and particles are not formed even after 40 m reaction time, indicating that EDA or IPA do not cause reduction of the Pt complex under these conditions. Furthermore, in the presence of NaBH_4_, solutions of PtCl_2_/EDA + NaBH_4_/H_2_O turn grey within 2 min and gradually become black after 40 min reaction time, due to the formation of Pt particles after reduction of the Pt complex by NaBH_4_. Also, solutions of PtCl_2_/EDA/IPA+ NaBH_4_/H_2_O turn grey after 5 min, indicating that the reduction rate is slower in the presence of IPA.

**Figure 6.**
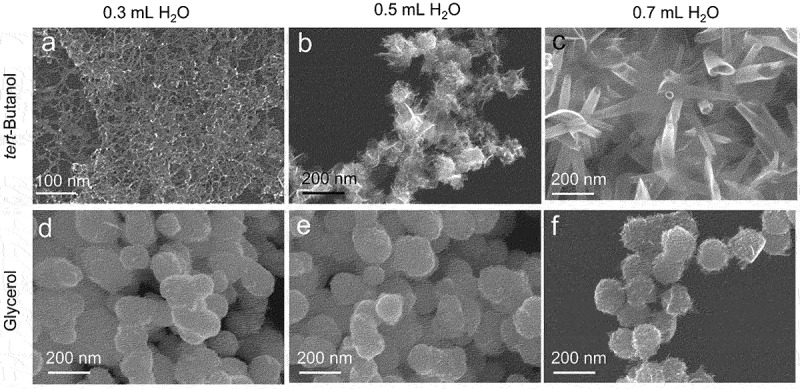
SEM images of synthesized Pt particles using *tert*-butanol at condition of (a) 0.3 mL H_2_O, (b) 0.5 mL H_2_O and (c) 0.7 mL H_2_O. SEM images of synthesized Pt particles using glycerol at condition of (a) 0.3 mL H_2_O, (b) 0.5 mL H_2_O and (c) 0.7 mL H_2_O.

As described in Figures 1 and 2, the morphologies of Pt NFs (Figure 1) are similar regardless of the volume of H_2_O used while the morphologies of Pt particles (Figure 2) vary according to the addition of IPA and H_2_O, revealing that IPA is important for controlling the morphology. To confirm the role of IPA, we replaced IPA with tert-butanol or glycerol at constant Pt: alcohol molar ratio, as shown in Figure 6. Using the same synthesis procedure, IPA was replaced with 4.3 mL tert-butanol (0.04 mol) or 3.3 mL glycerol (0.04 mol) added to 2 mL Pt/EDA precursor. In the case of *tert*-butanol, a similar result was obtained. That is, PtNWs, PtNSs and PtNTs were observed, respectively, for 0.3, 0.5, and 0.7 mL of H_2_O (Figure 6(a–c)). However, glycerol cannot be used effectively to regulate the morphology with spherical particles produced with different volumes of H_2_O added (Figure 6(d–e)). Both IPA and *tert*-butanol have similar structures containing alkyl and hydroxyl groups, whereas glycerol contains multiple hydroxyl groups and no hydrophobic alkyl group. This suggests that the alkyl group of the alcohol molecules plays an important role in regulating the morphology possibly by establishing amphiphilic effects in the aggregate precursors of the Pt particles.

**Figure 7.**
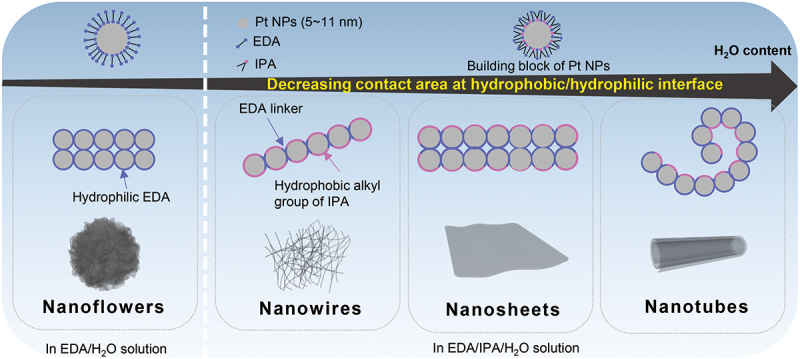
Schematic illustrating the self-assembly strategy for the synthesis of metallic Pt nanoflowers, nanowires, nanosheets and nanotubes.

Based on the above experimental results, we here propose a possible mechanism to explain the formation of the different morphologies of Pt particles obtained. We speculate that the initial small Pt NPs (5-11 nm   ) formed during reduction by NaBH_4_ are stabilized in aqueous solution by adsorption of EDA molecules. EDA molecules adsorb at the surface of Pt below 340 K forming weak chemical bonds between lone pairs of its N atoms and Pt atoms at the surface [43,44]. In solution, the adsorption and desorption of EDA molecules at the metal surfaces is in dynamic equilibrium [45] so that EDA molecules continuously detach or reattach at the Pt NPs surfaces during the assembly process. The adsorption of EDA molecules increases the hydrophilicity of the Pt NPs because the amino group has polar character and stabilizes Pt NPs in the solution. In EDA/H_2_O solution, as shown on the left of the dashed line in Figure 7, EDA not only stabilizes but can also connect Pt NPs due to the presence of two amino groups [46]. The formation of the sheet structure observed in PtNFs can then be explained as being due to a minimization of surface energy by increasing the interfacial area between EDA on PtNFs and H_2_O solvent. Subsequently, Pt-EDA sheets are considered to co-aggregate through the EDA ligands to form PtNFs. On the other hand, in the presence of IPA, the hydrophobic effect of the alkyl group of IPA appears as shown to the right of the dashed line in Figure 7. It is known that IPA adsorbs on Pt through interactions involving hydroxyl groups so that the surface hydrophobicity of Pt NPs will increase compared to the Pt-EDA species. The morphological variation of Pt particles from nanowires, nanosheets, to nanotubes, observed simply by changing the H_2_O content is explained as being a result of reducing the contact area between hydrophobic Pt particles and H_2_O solvent [47,48]. The self-assembly behavior of EDA/IPA-stabilized Pt NPs is thus similar to that of the surfactant-promoted self-assembly processes, suggesting that Pt NPs assembly might also involve formation of amphiphilic surfaces. Our proposed mechanism also accounts for the formation of PtNTs obtained from curling nanosheets as the H_2_O content increases.

## 4. Conclusions

Controllable synthesis of Pt particles with various morphologies has been achieved by a simple chemical reduction method using EDA, IPA and H_2_O, where varying the relative proportion of H_2_O can be used to regulate the behaviors of Pt NPs-based building blocks during self-assembly. Structural characterization reveals that different morphologies of Pt particles, including nanoflowers, nanowires, nanosheets and nanotubes, are obtained. We propose a growth model that involves self-assembly of the initially formed Pt NPs through EDA linker molecules, with control of the hydrophilic/hydrophobic interactions according to IPA and H_2_O contents. The Pt particles prepared here show improved ORR activity over Pt black, which can be explained by considering the effects of their hierarchical structures. Overall, our results establish that a facile self-assembly synthesis of Pt particles with nanoarchitectonic morphological control can be applied for the development of highly active Pt catalysts. This work not only presents the morphological diversity of Pt particles available by using self-assembly methods, but also establishes the nanoarchitectonics concept for the construction of hierarchical morphologies of Pt using Pt NPs as building blocks.
